# Exposure to a Low Pathogenic A/H7N2 Virus in Chickens Protects against Highly Pathogenic A/H7N1 Virus but Not against Subsequent Infection with A/H5N1

**DOI:** 10.1371/journal.pone.0058692

**Published:** 2013-03-04

**Authors:** Júlia Vergara-Alert, Ana Moreno, Juliana G. Zabala, Kateri Bertran, Taiana P. Costa, Iván Cordón, Raquel Rivas, Natàlia Majó, Núria Busquets, Paolo Cordioli, Fernando Rodriguez, Ayub Darji

**Affiliations:** 1 Centre de Recerca en Sanitat Animal, Universitat Autònoma de Barcelona- Institut de Recerca i Tecnologia Agroalimentàries, Campus de la Universitat Autònoma de Barcelona, Bellaterra (Cerdanyola del Vallès), Spain; 2 Istituto Zooprofilattico Sperimentale della Lombardia e dell’Emilia Romagna, Brescia, Italy; 3 Departament de Sanitat i Anatomia Animals, Universitat Autònoma de Barcelona, Bellaterra (Cerdanyola del Vallès), Spain; 4 Institut de Recerca i Tecnologia Agroalimentàries, Barcelona, Spain; St. Jude Children’s Research Hospital, United States of America

## Abstract

Recent evidences have demonstrated that the presence of low pathogenic avian influenza viruses (LPAIV) may play an important role in host ecology and transmission of avian influenza viruses (AIV). While some authors have clearly demonstrated that LPAIV can mutate to render highly pathogenic avian influenza viruses (HPAIV), others have shown that their presence could provide the host with enough immunological memory to resist re-infections with HPAIV. In order to experimentally study the role of pre-existing host immunity, chickens previously infected with H7N2 LPAIV were subsequently challenged with H7N1 HPAIV. Pre-infection of chickens with H7N2 LAPIV conferred protection against the lethal challenge with H7N1 HPAIV, dramatically reducing the viral shedding, the clinical signs and the pathological outcome. Correlating with the protection afforded, sera from chickens primed with H7N2 LPAIV reacted with the H7-AIV subtype in hemagglutination inhibition assay and specifically with the N2-neuraminidase antigen. Conversely, subsequent exposure to H5N1 HPAIV resulted in a two days-delay on the onset of disease but all chickens died by 7 days post-challenge. Lack of protection correlated with the absence of H5-hemagglutining inhibitory antibodies prior to H5N1 HPAIV challenge. Our data suggest that in naturally occurring outbreaks of HPAIV, birds with pre-existing immunity to LPAIV could survive lethal infections with HA-homologous HPAIV but not subsequent re-infections with HA-heterologous HPAIV. These results could be useful to better understand the dynamics of AIV in chickens and might help in future vaccine formulations.

## Introduction

Avian influenza viruses (AIV) can be classified into low (LPAIV) and high (HPAIV) pathogenic avian influenza viruses depending on the severity of the disease that they cause, which ranges from asymptomatic infection to acute systemic disease and even death [1]. During the last decades, HPAIV have been involved in several outbreaks in poultry and wild birds around the world. The disease has had a severe economic impact because millions of birds died or have been killed to prevent the spread of the virus [2]. Seventeen HA and 9 NA subtypes have been identified so far [3], [4] but HPAIV have been **only** described for the H5 and H7 subtypes.

It is well known that LPAIV can mutate into HPAIV. An example occurred during the outbreak in 1999–2000 in Italy. The isolated virus was first characterized as an H7N1 LPAIV, but some months later an H7N1 HPAIV causing 100% of mortality was isolated in a turkey flock [5]. On the other hand, HPAIV could also appear as a consequence of reassortments between different LPAIV subtypes that co-infect wild birds, their natural reservoirs [6], [7]. Therefore, it seems important that surveillance programs should focus on the control of LPAIV, mainly those caused by viruses of the H5 or H7 subtypes, to prevent future emergences of HPAIV [8]. Although the virulence can be linked to the presence of multiple basic amino acids in the hemagglutinin (HA) cleavage site, the acquisition of a multibasic cleavage site alone can be insufficient to increase viral pathogenicity [9].

Conversely to the inherent risks of their presence, pre-existing immunity due to LPAIV have also been demonstrated to confer a certain degree of protection against subsequent challenges with LPAIV and HPAIV in different species [10], [11], [12], [13], [14], [15]. To characterize the impact of pre-existing immunity, chickens were experimentally **infect** to assess whether the pre-exposure to H7N2 LPAIV can confer protection against H7N1 HPAIV and also, against a subsequent challenge with H5N1 HPAIV. Pre-infection of chickens with H7N2 LPAIV **conferred protection** against a secondary infection with HA-homosubtypic HPAIV. However, surviving chickens did not resist subsequent infection with a lethal dose of the HA-heterosubtypic HPAIV, with only a slight delay on the disease outcome. The protection status directly correlated with the presence in the sera of hemagglutinin inhibitory antibodies against the specific HA-subtype.

## Materials and Methods

### Ethics Statement

The present study was performed in strict accordance with the Guidelines of the Good Experimental Practices. Animal procedures were approved by the Ethical and Animal Welfare Committee of *Universitat Autònoma de Barcelona* (UAB) (Protocol #DMAH-5767). Chicken experiments were conducted at Biosafety Level 3 (BSL-3) facilities of the *Centre de Recerca en Sanitat Animal (CReSA*-Barcelona).

### Influenza Viruses

The viruses used in this study were the LPAIV A/*Anas plathyrhynchos*/Spain/1877/2009 (H7N2), the HPAIV A/FPV/Rostock/34 (H7N1) and the HPAIV A/Great crested grebe/Basque Country/06.03249/2006 (H5N1). The H7N2 LPAIV strain was obtained from the ongoing surveillance program carried out in Catalonia, north-east Spain. The H7N1 HPAIV was generated by reverse genetics, as reported previously [16] and the H5N1 HPAIV virus was isolated from a surveillance program in north-Spain [17].

Virus stocks were propagated in the allantoic fluid of 11-day-old specific pathogen free (SPF) embryonating chicken eggs at 37°C for 48 h (H5N1 HPAIV) and for 72 h (H7N2 LPAIV and H7N1 HPAIV). The allantoic fluids were harvested, aliquoted and stored at −80°C until use. The infectious virus titre was determined in SPF embryonating chicken eggs and titres were measured as median embryo infectious dose (EID_50_) for H7N2 LPAIV and median embryo lethal dose (ELD_50_) for H7N1 and H5N1 HPAIV by following the Reed and Muench method [18].

### Animals and Experimental Design

Thirty SPF chicken eggs (Lohmann Tierzucht GmbH, Germany) were hatched under BSL-3 containment conditions at CReSA. At 2-week-old, chicks were divided into three groups (*Table 1*). Each group was housed in independent biocontainment isolation units ventilated under negative pressure with high efficiency particulate air filters. **Birds in group** 1 (G1; n = 10) were initially inoculated with H7N2 LPAIV (10^5.5^ EID_50_/50 µl) and challenged 15 days later with H7N1 HPAIV (10^5.5^ ELD_50_/50 µl). Two weeks after the H7N1 HPAIV challenge, six animals from group 1 were inoculated with H5N1 HPAIV (10^4.5^ ELD_50_/50 µl). **Birds in group** 2 (G2; n = 10) were inoculated with saline solution and challenged two weeks later with H7N1 HPAIV (10^5.5^ ELD_50_/50 µl): this group served as positive control of H7N1 HPAIV infection. Finally, **birds in group** 3 (G3; n = 10) were inoculated with saline solution twice at a 15-day interval; two weeks later, six animals from this group were inoculated with H5N1 HPAIV (10^4.5^ ELD_50_/50 µl). This group served as a positive control of H5N1 HPAIV infection. All animals received the inoculums intranasally and in a volume of 50 µl.

**Table 1:** Experimental design.

Group	N°animals (n)	Inoculum 1 Day 0	Inoculum 2 Day 15	N°animals (n)	Inoculum 3 Day 30
G1	10	H7N2 LPAIV^a^	H7N1 HPAIV^b^	6	H5N1 HPAIV^c^
G2	10	Saline solution	H7N1 HPAIV	–	–
G3	10	Saline solution	Saline Solution	6	H5N1 HPAIV

Thirty 2-week old SPF-chickens were randomly distributed into three groups. Animals received the first inoculum (day 0) and 2 weeks later (day 15), birds were challenged with the respective inoculum 2. Six birds from G1 and G2 were consecutively infected 2 weeks later (day 30) with the third inoculum.

Abbreviations: LPAIV = low pathogenic avian influenza virus; HPAIV = highly pathogenic avian influenza virus.

a Chickens from G1 were inoculated intranasally with LPAIV A/*Anas plathyrhynchos*/Spain/1877/2009 (H7N2) (10^5.5^ ELD_50_). Birds from G2 and G3 received saline solution.

b Chickens from G1 and G2 were intranasally challenged with HPAIV A/FPV/Rostock/34 (H7N1) (10^5.5^ ELD_50_) 15 days after the pre-exposure to H7N2 LPAIV. Birds from G3 received saline solution.

c Chickens from G1 and G3 were inoculated intranasally with 10^4.5^ ELD_50_ of A/Great crested grebe/Basque Country/06.03249/2006 (H5N1) 15 days after the challenge with H7N1 HPAIV.

Chickens were monitored for the development of any flu-like clinical signs, and the mean clinical score and mortality rate (mean death time, MDT) were recorded. The intensity of the clinical signs was assessed by a semi-quantitative scoring: healthy (0), sick (1), severely sick (2), moribund or dead (3). **Animals classified as “sick” were those birds showing one of the following signs: respiratory involvement, depression, diarrhea, cyanosis of the exposed skin or wattles, edema of the face and/or head, nervous signs. Birds were given a score of 2 (“severely sick”) when showing more than one of the mentioned signs.** According to ethical procedures, animals presenting severe clinical symptoms (score 2) were euthanized with intravenous administration of sodium pentobarbital (100 mg/kg, Dolethal®, Vétoquiunol, France).

For the serological analysis, blood was collected from all birds 15 days post-H7N2 LPAIV infection and 10 days after H7N1 HPAIV infection. In addition, cloacal (CS) and oropharyngeal (OS) swabs were collected for virus isolation at 1, 4, 7, and 12 days post-H7N2 LPAIV inoculation, and at 1, 4, 7, and 12 days after H7N1 HPAIV challenge. The experiment was terminated 10 days after H5N1 HPAIV inoculation, time at which all the remaining birds were euthanized as described above and full necropsies were performed. All samples were stored at −80°C until tested.

### Histopathology

Necropsies and tissue sampling were performed according to a standard protocol. For histopathological analysis, samples of central nervous system, heart, kidney, spleen, thymus, bursa of Fabricius and liver were **immediately** fixed in 10% neutral buffered formalin, dehydrated and embedded in paraffin. **Tissue sections were processed routinely for hematoxylin/eosin (H/E) staining.**

### Virus Quantification by Real Time RT-PCR (RRT-PCR)

Viral RNA quantification using one step RRT-PCR was performed in OS and CS, which were collected in sterile Dulbecco’s modified Eagle’s medium (DMEM) (Life Technologies, S.A., UK) with antimicrobial drugs (100 units ml^−1^ penicillin-streptomycin). Viral RNA was extracted with QIAamp Viral Mini kit (Qiagen, Inc., Germany). Amplification of a matrix gene fragment was carried out using primers, probe, One-Step RT-PCR Master Mix Reagents (Life Technologies, S.A, UK) as previously reported [19] and amplification conditions as described by Busquets and collaborators [20] in Fast7500 equipment (Life Technologies, S.A, UK) using 5 µl of eluted RNA in a total volume of 25 µl. The limit of the detection of the assay was six viral RNA copies of *in vitro*-transcribed RNA per reaction, which was equivalent to Ct = 39.16.

### Solid Phase Competitive ELISA for H7-antibody Detection

A competitive ELISA was developed for the evaluation of the presence of specific H7-antibodies in serum samples as previously described [21]. Briefly, micro-plates (Nunc, MaxiSorp™ microplates, DK, US) were coated with 50 µl per well of H7 AIV antigen diluted 1∶500 in coating buffer (sodium bicarbonate 0.1 M) overnight at 4°C. The LPAIV [A/Turkey/Italy/2676/99 (H7N1)] used as antigen was previously clarified, inactivated with β-propiolactone and partially purified by ultracentrifugation through a 25% (w/w) sucrose cushion. Sera from individuals were added to the H7 AIV-coated plate with 10-fold dilutions (starting from 1∶10) and 25 µl of anti-H7 horseradish peroxidase (HRP)-conjugated monoclonal antibody (MAb) (7A4) were immediately added. After 1 h incubation at 37°C, the plates were washed three times (PBS 1×/0.1% Tween20) and 50 µl of activated o-Phenylenediamine dihydrochloride (OPD) substrate solution were added to the wells. After 10 min incubation at room temperature (RT) the optical density (OD) was measured at 492 nm. Positive H7N1 anti-serum (HI titre: 8 log_2_) and negative control serum were included in each plate.

### Liquid-phase Blocking ELISA (LPBE) for N1- and N2-antibody Detection

Sera were analyzed for the presence of N1 and N2 antibodies as previously described [22]. Briefly, 96-well plates (Nunc, MaxiSorp™ microplates, DK, US) were coated with 50 µl per well of N1- (5B2, diluted 1∶500) or N2- (4C11, diluted 1∶200) specific capture MAbs in coating buffer (sodium bicarbonate 0.1 M) overnight at 4°C. AIV used as antigens in the respective LPBE [A/goose/Italy/296426/03 (H1N1) LPAIV and A/Turkey/England/28/73 (H5N2) LPAIV] were previously inactivated with β-propiolactione and then disrupted by adding Triton X100 to a final concentration of 3%. Mixtures of antigen at a pre-determined dilution and test sera diluted 1/2 and 1/4 (1/4 and 1/8 final dilutions) were pre-incubated at 37°C for 60 min in an auxiliary micro-plate, then 50 µl were transferred into the respective MAb-coated plate and further incubated at 37°C for 60 min. Plates were washed three times with PBS 1×/0.1% Tween20 and 50 µl of the homologous anti-N1 (5B2) and anti-N2 (4C11) HRP-conjugated MAb was added to wells followed by 1 h incubation at 37°C. After washing the plates three times (PBS 1×/0.1% Tween 20), 50 µl of OPD substrate solution were added to the wells and allowed to develop for 8–10 min at RT. The OD was measured at 492 nm. An H7N1 anti-serum (HI titre: 8 log_2_) and H9N2 anti-serum (HI titre: 8 log_2_) were used as positive controls in the N1- and N2-ELISA, respectively. Serum from SPF chickens was used as a negative control.

Results from both ELISAs were calculated by determining the absorbance value reduction and were expressed as percentage of inhibition with respect to the reference value (100% control wells).

### Hemagglutination Inhibition Test

Serum samples were also analyzed for the presence of antibodies against specific H5- and H7-subtypes by hemagglutination inhibition (HI) test. The assay was performed according to the international standard procedure [23] for testing avian sera using chicken red blood cells and 4 hemagglutination units of either H5N1 or H7N2 AIV. To avoid nonspecific positive reactions, sera were pre-treated by adsorption with chicken red blood cells and heat-treated at 56°C for 30 min. Known positive and negative sera were used as controls.

### Statistical Analysis

Data obtained from the evaluation of OS and CS by RRT-PCR were analyzed by Kruskal-Wallis test for significant differences (*p*<0.05) between groups. The statistical tests were performed using the Statistical Package for the Social Sciences (SPSS) for Windows Version 17.0.

## Results

### Pre-exposure to LPAIV Protects against the Infection with an HA-homosubtypic HPAIV

**Figure 1:**
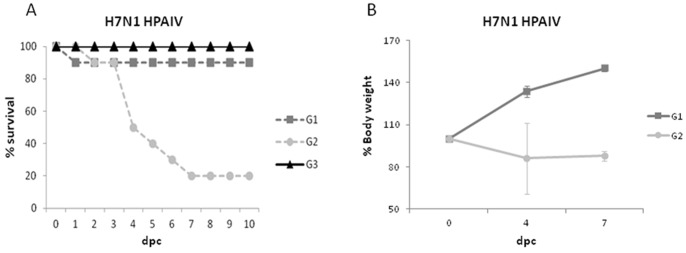
Lethality and weight loss in chickens after challenge with H7N1 HPAIV. (A) Survival curves (in percentage) of SPF-chickens from G1 (pre-exposed to H7N2 LPAIV), G2 (positive control) and G3 (negative control) after H7N1 HPAIV-challenge. (B) Weight loss curves of SPF-chickens from G1 and G2 after infection with H7N1 HPAIV. Mean %-body weight of animals normalized to initial weight ± SD is represented.

In order to assess the role of pre-existing immunity in subsequent HPAIV infections, SPF-chickens were experimentally inoculated with H7N2 LPAIV and 15 days later challenged with H7N1 HPAIV (the same HA-subtype). No clinical signs or lesions were observed after H7N2 LPAIV inoculation (G1), whereas inoculation of naïve animals with H7N1 HPAIV (G2) induced severe clinical signs and mortality from day 2 after inoculation (*Figure 1A*). Clinical signs mainly consisted in depression, apathy and ruffled feathers. Impaired breathing was observed in some of the animals from G2. Mortality was recorded until 7 days post-inoculation (dpi) and MDT was 4.5 days (range 2–7 days). In clear contrast, chickens pre-infected with H7N2 LPAIV were effectively protected against H7N1 HPAIV challenge. Thus, nine out of ten chickens from G1 survived, showing only ruffled feathers at 1 dpi and no additional clinical signs of disease. The only animal from G1 that died at 1 dpi did not show flu-like clinical signs or pathological lesions. Additionally, birds from this group gained weight normally, while G2-birds lost it (*Figure 1B*).

After H7N1 HPAIV-challenge, lesions related to influenza were observed only in G2 from 3 days post-challenge (dpc) onwards. At 3 dpc, petechial hemorrhages on the comb and leg edema were present only in one bird. Hemorrhages on the comb, wattles and legs were present in almost all the animals (8/10) from 4 dpc onwards. Between day 4 and 6 after challenge, crop congestion and multiple petechia in the proventricular mucosa were detected in almost all birds (7/10). No lesions were observed in G1 confirming the solid protection against H7N1 HPAIV by the pre-exposition to H7N2 LPAIV. Animals from G3 (sham inoculated group) did not show clinical signs or lesions during this period **(data not shown).**

### Previous Infections with LPAIV and HPAIV do not Protect Against Subsequent Challenge with an HA-heterosubtypic HPAIV

**Figure 2:**
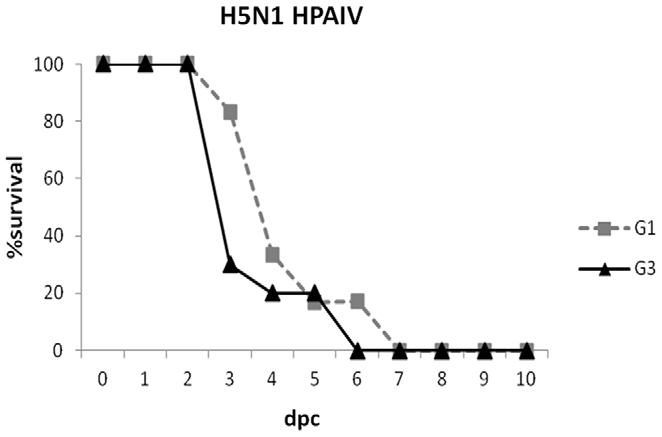
Lethality in chickens after challenge with H5N1 HPAIV. Survival curves (in percentages) of SPF-chickens from G1 (pre-exposed to H7N2 LPAIV and subsequently infected with H7N1 HPAIV) and G3 (positive control) after H5N1 HPAIV-challenge.

To further analyze the potential cross protection afforded by the successive infection, two weeks after H7N1 HPAIV challenge, six chickens from G1 were inoculated with H5N1 HPAIV. Six birds from G3 were used as H5N1 HPAIV-positive control. Three days after H5N1 HPAIV-challenge, one chicken (16.6%) from G1 died while five from G3 (83.3%) succumbed. In spite of this apparent delay of mortality rate, only one of the birds from G1 remained alive by 5 dpc and all were dead by 7 dpc (*Figure 2*). All animals lost weight and either exhibited neurologic signs prior to succumb or were found dead without previous clinical manifestations. The onset of morbidity ranged from 2 to 5 dpc in birds from G3 and from 3 to 6 dpc in G1. For the control group (G3) MDT was 3.7 days (range 3–6 days), while in G1 MDT was 4.5 days (range 3–7 days).

### Previous Infection with LPAIV Reduces HPAIV Shedding

**Figure 3:**
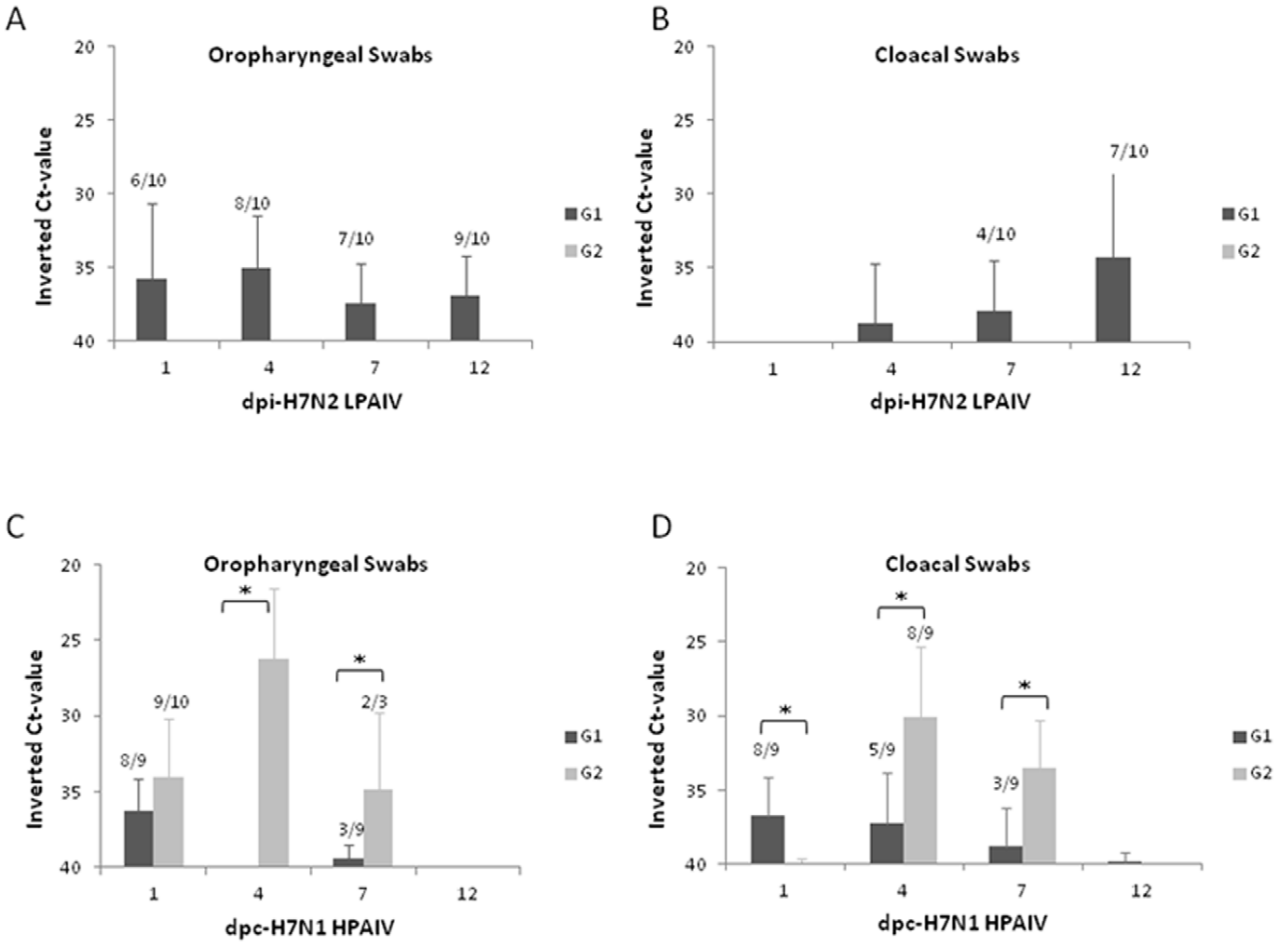
Viral shedding from experimental infected chickens with H7N2 LPAIV and to subsequent infection with H7N1 HPAIV. Viral RNA shedding measured by RRT-PCR in swab samples (oropharyngeal and cloacal) at 1, 4, 7 and 12 days after (A) H7N2 LPAIV infection and (B) H7N1 HPAIV challenge. Results are expressed as inverted Ct-value and shown as means ± SD. The number of animals shedding/total birds is indicaed, except in those cases that the sheddin was 100%. Ct, cycle of threshold. Asterisk (*) indicates statistical significant differences (*P*<0.05).

Oropharyngeal and cloacal shedding was assessed on days 1, 4, 7 and 12 after H7N2 LPAIV inoculation and H7N1 HPAIV challenge. After H7N2 LPAIV-inoculations, all chickens from G1 showed viral shedding at least once during the selected time-points as detected in either the OS, CS or both. No viral RNA was detected in G2 which, at this time-point, only received saline solution (*Figure 3A, 3B*). After H7N1 HPAIV-infection, all chickens from G2 (exposed only to H7N1 HPAIV) showed a consistent viral shedding from 1 to 7 dpc. No viral RNA was detected at 12 dpc in the two animals that survived. Conversely, pre-exposure to H7N2 LPAIV significantly (*p*<0.05) reduced shedding of H7N1 HPAIV from 4 dpc onwards, as compared to positive controls (G2) (*Figure 3C, 3D*). Although in G1 viral RNA was detected at 1 dpc (in both OS and CS), it is not possible to confirm whether the viral RNA detected is from H7N1 HPAIV or form the previous H7N2 LPAIV inoculation.

### Pre-existing Immunity to AIV has a Role in the Outcome of HPAIV Infection

Sera collected from chickens that were pre-exposed to H7N2 LPAIV (G1) inhibited hemagglutination by H7N3 antigen but did not elicit HI titers against H5N1 antigen (*Table 2*). Serum from only one animal from this group did not show any H7-hemagglutination inhibitory activity. However, it did not show clinical signs after H7N1 HPAIV infection. Sera collected 10 days after H7N1 HPAIV infection also inhibited the hemagglutination by H7N3 in all the birds from G1 and in the two birds from G2 that survived until the end of the experiment (*Table 2*).

**Table 2:** Serological status, as determined by hemagglutination inhibition, of chickens 15 days after experimental pre-exposure to H7N2 LPAIV^a^ and 10 days after challenge with H7N1 HPAIV^b^.

	HI Titer^c^				
Group Bird identification		15 days post-H7N2/LP exposure (Day 15)		10 days post-H7N1/HP challenge (Day 25)	
		H7^d^	H5^e^	H7	H5
**G1**					
1		16	<4	32	<4
2		32	<4	64	<4
3		8	<4	8	<4
4		<4	<4	32	<4
5		64	<4	128	<4
6		32	<4	64	<4
**G2**					
7		<4	<4	†	†
8		<4	<4	†	†
9		<4	<4	†	†
10		<4	<4	128	<4
11		<4	<4	†	†
12		<4	<4	128	<4
**G3**					
13		<4	<4	<4	<4
14		<4	<4	<4	<4
15		<4	<4	<4	<4
16		<4	<4	<4	<4

Sera from the animals were tested against H7 and H5 antigens.

a Chickens were inoculated intranasally with A/*Anas plathyrhynchos*/Spain/1877/2009 (H7N2) (10^5.5^ ELD_50_). Serologic data from six randomly selected birds per group are presented.

Due to the lack of seroconversion, only four animals from the naïve group are represented in the table.

b Chickens were challenged intranasally with A/FPV/Rostock/34 (H7N1) (10^5.5^ of ELD_50_) 15 days after the pre-exposure to H7N2 LPAIV.

c HI titers ≥8 were considered positive.

d HI using antigen against H7N3 subtype.

e HI using antigen against H5N1 aubtype.

† = succumbed to H7N1 HPAIV-infection.

**Figure 4:**
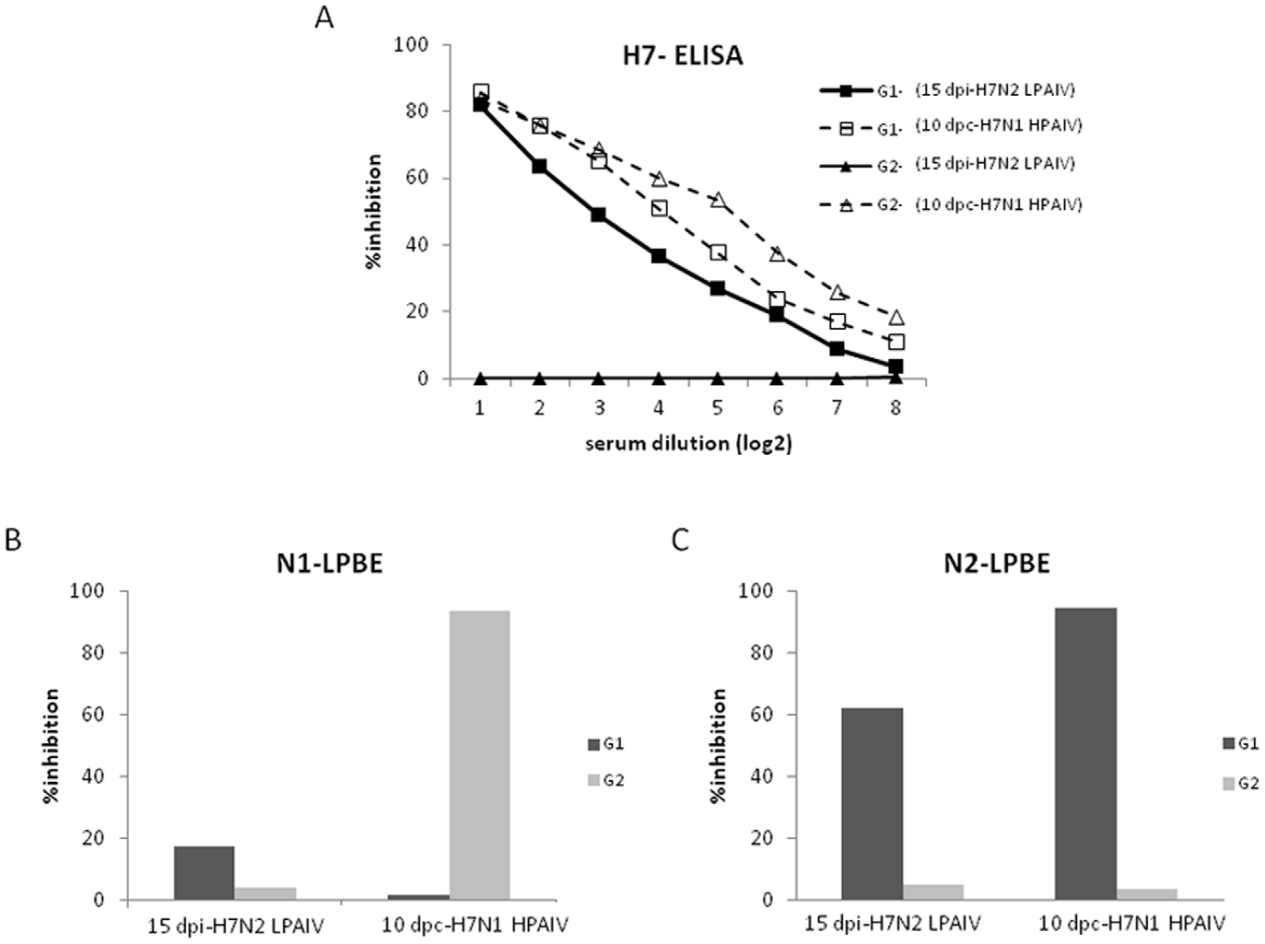
Presence of specific antibodies against H7-, N1- and N2- evaluated by ELISA. Pooled sera from chickens were taken 15 days post-H7N2 LPAIV exposure and 10 days post-H7N1 HPAIV challenge and were tested for binding to (A) H7 hemagglutinin, (B) N1 or (C) N2 neuraminidases by ELISA.

To further characterize the elicited humoral response, sera were also analyzed for the presence of antibodies against the specific hemagglutinin (H7) and neuraminidases (N2 or N1) by ELISA (*Figure 4*). As expected, the specific HA-ELISA yielded similar results than the HI assay (*Table 2*). Interestingly, no significant boosting effect was observed for the G1 group after H7N1 HPAIV challenge. Moreover, lower titers of antibodies against the H7-hemagglutinin seemed to exist for animals within this group than for the two survivors from the G2 at a given time (*Figure 4A*). In agreement with this data, sera from G1 elicited specific anti-N2 antibodies (*Figure 4C*) but did not elicit specific antibodies against N1, even after H7N1 HPAIV infection (*Figure 4B*). In contrast, sera from survivor chickens from the G2 showed antibodies against N1 but not against N2.

## Discussion

Avian influenza (AI) is still an important challenge for the scientific community. **Although most efforts** have been focused in studying the disease and the etiological agent, the potential of AIV to cause a pandemic is constant. Therefore, AI remains as an important matter for the ecology, the economy and the public health. AIV from the H5- and H7- subtypes are extremely susceptible to accept pathogenic mutations such as polybasic **amino acids** in the HA-cleavage site [24]. According to the presence of the polybasic motive in their sequence, both the H7N1 HPAIV and the H5N1 HPAIV used in this study **are** highly pathogenic. H7N1 HPAIV caused 80% mortality and H5N1 HPAIV provoked 100% mortality in naïve SPF-chickens.

It is important to include the detection and control of LPAIV in surveillance programs because it has been clearly established that HPAIV have their origin in circulating LPAIV through mutation [25] and/or genetic reassortments between LPAIV of different subtypes concomitantly infecting wild reservoirs [6], [7]. On the other hand, surveillance work have also demonstrated that immunological memory printed by circulating LPAIV could also **be** beneficial for the host, since **it** can confer certain degree of protection against circulating viruses species [10], [11], [12], [13], [14], [15]. **However, this protection conferred by a LPAIV against HPAIV could also mask low levels of HPAI in a flock and this can generate an outbreak in naïve populations.**

As described for other LPAIV strains in domestic and wild birds [10], [25], in the present study, inoculation of the H7N2 LPAIV protected chickens from HA-homosubtypic challenge (H7N1 HPAIV) and this protection coincided with the presence of specific **hemagglutinin** inhibitory antibodies prior to challenge. Interestingly enough, human vaccines only protect against closely related viruses and do not confer protection to all viruses sharing same HA-subtype. Thus, the protection afforded depends on the antigenic match between the viruses in the vaccine and those circulating [26].

**Although there is a correlation between the presence of anti-H7 antibodies and protection, there was one animal that resulted protected in the absence of detectable antibodies prior to FPV challenge.** Several mechanisms could explain the protection afforded in this bird, including the induction of cross-reactive T-cells [27], [28], [29]. The presence of low, albeit undetectable levels of H7-inhibitory antibodies before HPAIV challenge in this animal should not be ruled out. This hypothesis seems to be confirmed by the fact that this single individual showed similar levels of H7-specific hemagglutinin inhibitory activity after HPAIV challenge, than the rest of the animals within the group, indicating the existence of some kind of previous priming (*Table 2*).

Lack of solid protection against H5N1 HPAIV challenge correlated again with the absence of anti-H5 antibodies prior to challenge. The slight delay found on disease onset observed for the G1 animals could be related, again, with the induction of cross-reactive T-cells or with the induction of cross-reactive antibodies against other viral determinants [30]. The fact that almost no anti-N1 antibodies were present in pre-immunized chickens seemed to rule out their implication in the protection observed, contrary to that observed in other studies in pigs [31]. The absence of protection against H5N1 HPAIV was somehow surprising taking into account recent published results using a similar experimental approach [13]. In this case, mallard ducks infected twice (21 days apart) with an H7N7 LPAIV were solidly protected against heterosubtypic challenge with a H5N2 LPAIV. The degree of protection observed between each of these studies was extremely variable and might depend on multiple factors including: the host, the strain and virulence of the AIVs used during the experimental procedure and the time-interval spanned between the infections [15].

Hetererologous protection is a goal to be achieved by almost all future vaccines, not only against influenza. Pre-exposure to H7N2 LPAIV confers protection against a subsequent infection with H7N1 HPAIV. The immune response induced by H7N2 LPAIV not only protected from H7N1 HPAIV mortality, clinical signs and viral shedding, but also blocked the incoming HPAIV to the point of not allowing enough antigen to prime for antibodies against the N1-neuraminidase, **or** to boost the anti-H7 antibodies. These data could also have important implications for the host ecology because, in case of subsequent infections, the transmission of the virus between animals, although present, would be reduced. **Although this can benefit a particular flock, it is important to assess the risk of masking an introduction of a HPAIV which can be spread among the flock and shed to other populations.**
